# Knowledge and Practices Regarding Deep Venous Thrombosis (DVT) Prevention Among Nurses in Jeddah, Saudi Arabia

**DOI:** 10.3390/nursrep14040289

**Published:** 2024-12-11

**Authors:** Ruba M. Alharazi, Raiannah H. Alqahtani, Rahaf A. Alanazi, Walaa Alharbi, Shmokh M. Alshenen, Aisha Alhofaian, Afnan Tunsi, Loujain Sharif

**Affiliations:** 1 Medical Surgical Nursing Department, Faculty of Nursing, King Abdulaziz University, Jeddah 21589, Saudi Arabia; 2 Faculty of Nursing, King Abdulaziz University, Jeddah 21589, Saudi Arabia; 3 Psychiatric and Mental Health Nursing Department, Faculty of Nursing, King Abdulaziz University, Jeddah 21589, Saudi Arabia

**Keywords:** deep vein thrombosis, nurses, knowledge, prevention, practice

## Abstract

Background/Objectives: Deep venous thrombosis (DVT), the formation of a blood clot within a large vein, is one of the most common problems among hospitalized patients. The annual prevalence of DVT is 48 per 1,000,000. Nurses’ knowledge significantly affects compliance with VTE risk assessment and prevention. This study aimed to assess the knowledge and practices regarding deep venous thrombosis prevention among nurses in Ministry of Health hospitals and King Abdulaziz University Hospital (KAUH), Jeddah, Saudi Arabia. Methods: This study was conducted in Jeddah using a quantitative, descriptive, cross-sectional design. A sample of 240 registered nurses were conveniently recruited to complete a self-administered online questionnaire. The data were coded and analyzed through SPSS version 24. Results: The participants had adequate knowledge on the prevention of DVT (75.64 ± 18.88), and the highest level was observed for knowledge about the prevention and prophylaxis of DVT (81.98 ± 45.73%). The practice level of nurses in preventing DVT was 71.92%, with a mean score of 18.7. Conclusions: There is a significant effect of nurses’ level of academic qualifications, working ward, and DVT prevention training on their knowledge and practice of DVT prevention.

## 1. Introduction

Deep venous thrombosis, the formation of a blood clot within a large vein, is one of the most common problems among hospitalized patients [1]. Three significant DVT manifestations are pain, swelling, and movement disorder [2]. Venous thromboembolism (VTE) represents a major public health issue on a global scale, with varying incidence rates across different regions. In Western nations, the annual incidence is reported to be between 100 and 269 cases per 100,000 individuals (Wendelboe & Raskob, 2016 [3]; Bénard et al., 2005 [4]). Conversely, in Eastern countries, the incidence is notably lower, falling below 100 cases per 100,000 person-years (Mahani et al., 2024 [5]). On a worldwide scale, the incidence of VTE ranges from 75 to 269 cases per 100,000 annually, with a significant increase observed in individuals aged 70 and older, where rates can reach between 200 and 700 per 100,000 (Raskob et al., 2014 [6]).

A study conducted in the Department of Surgery, University of Putra, Malaysia, aiming to determine whether the condition was indeed rare and whether the routine prophylactic measures as recommended by internationally accepted guidelines should be adopted in Asia, revealed that the prevalence of postoperative DVT ranges from 10% to 63% in Asia [7].

DVT is linked to a range of risk factors such as prolonged immobility, hospitalization, cancer, pregnancy, cardiovascular diseases, obesity, and recent surgical procedures (McLendon & Attia, 2017 [8]).

Prolonged bed rest is the most significant risk factor for developing DVT (OR = 1.612, 95% CI 1.090–2.385); this was addressed by a study aiming to explore whether risk factors for deep vein thrombosis are affected by bed rest durations and to identify different risk factors in groups with different bed rest durations, although bed rest, increased age, being in a surgical department, the female sex, and smoking are all considered essential risk factors [9].

One study aimed to gain insight into nursing resources in China and explore the relationship between nursing resources and the prevalence of major immobility complications among bedridden patients. The major immobility complications in the study were pressure ulcers, deep vein thrombosis, pneumonia, and urinary tract infection. DVT had a prevalence rate of 0.81% among hospitalized patients, which was no less than the prevalence of pressure ulcers, at 0.77% [10]. One significant and potentially fatal complication of DVT is pulmonary embolism (PE) [11]. The average annual prevalence of PE among both sexes is 69 per 100,000 [12].

Another study conducted in King Fahd General Hospital, Jeddah, Kingdom of Saudi Arabia, which aimed to determine the prevalence of confirmed VTE mortality among all-cause hospital mortalities, found that venous thromboembolism (VTE) accounted for 1.9% of all hospital deaths from 1 July 2008 to 30 June 2009 [13].

Another study was conducted in Jeddah at King Abdulaziz University Hospital to investigate the prevalence of deep vein thrombosis (DVT) in all adult patients who were suspected of having DVT and scanned by means of Doppler ultrasound in the Department of Vascular Imaging (DVI) at King Abdulaziz University Hospital (KAUH), Jeddah, over three years. The study revealed a significant increase in the prevalence of DVT in Jeddah (15.7%), and out of a total of 1201 patients diagnosed with DVT, 708 (58.2%) were female [14].

Besides all the medical treatments and prevention strategies, physical activity remains a significant prophylaxis strategy for DVT. A systemic review to assess the benefits or risks of physical activity in patients with an acute or previous DVT of the leg revealed that six months of daily exercises in patients with acute DVT were associated with vein recanalization and improved quality of life.

Deep vein thrombosis is a common vascular disorder among hospitalized patients, primarily bedridden intensive care unit (ICU) patients. If not appropriately prevented, it most likely leads to mortality and morbidity [13].

Although nurses are responsible for performing risk assessments and other preventive measures and are the first line in providing care for patients, a lack of knowledge on DVT prevention is a barrier to achieving effective nursing care [15].

Therefore, nurses’ knowledge must be assessed to ensure the best quality of healthcare and decrease the prevalence of DVT. A search of the literature revealed that few studies have been conducted in Jeddah to assess clinical nurses’ knowledge and practices regarding DVT prevention. Therefore, this study aimed to assess nurses’ knowledge and practices regarding deep vein thrombosis (DVT) prevention in Jeddah, Saudi Arabia. 

## 2. Materials and Methods

### 2.1. Study Design

This study used a quantitative, cross-sectional design. Data were collected at one specific point in time. This design is relatively fast and provides a snapshot of the conditions in a country at a specific time [6].

### 2.2. Study Setting and Participants

This study was conducted by means of a self-administered online questionnaire distributed in three Ministry of Health hospitals and King Abdulaziz University Hospital (KAUH) in Jeddah. The investigation started from 10 January 2023 to 13 February 2023.

Participants were selected via convenience sampling from the selected departments at the three MOH Hospitals and KAUH. The inclusion criteria were registered nurses, nurses of both sexes, nurses of all nationalities, nurses who consented to participate in this study, and nurses working in the following departments: medical ward, surgical ward, emergency, OB-GYN, ICU, CCU, and outpatient clinics at the Ministry of Health hospitals and King Abdulaziz University Hospital (KAUH) in Jeddah. Nursing students and nursing interns were excluded from this study.

Convenience sampling was used to recruit participants who met the inclusion criteria. The study sample consisted of 240 registered nurses working in Jeddah’s Ministry of Health hospitals or King Abdulaziz University Hospital (KAUH). Initially, the estimated sample size was 363, established through the Roasoft application, with a 5% margin of error and 95% confidence level.

### 2.3. Data Collection Method

The data were collected using an online self-administered questionnaire that was adopted from a study conducted by Yohannes S. et al. (2022) in Amhara Region Comprehensive Specialized Hospitals, northwest Ethiopia [16]. The questionnaire contains three parts: socio-demographic characteristics of respondents and other general details, knowledge level of nurses on the prevention of DVT, and practices of nurses in DVT prevention.

The first part of the questionnaire contains six multiple-choice questions regarding sex, current academic qualifications, work experience, hospital, and ward. The last three questions seek yes/no responses regarding whether participants have previous knowledge of DVT and a protocol/guideline in hospital settings.

Participants’ knowledge regarding DVT prevention and practice was assessed by the second part of the questionnaire, which has three sections: 2.1—nurses’ knowledge; 2.2—prevention and prophylaxis of DVT; and 2.3—risk factors of DVT. Questions in the second part comprise statements about nurses’ knowledge of DVT, the prevention and prophylaxis of DVT, and risk factors of DVT, which the participants must answer with true/false/don’t know.

The last part of the questionnaire concerns nurses’ practices in DVT prevention. The questions in this section present a situation or an event and asks participants whether they have practiced such an action. These questions are answered on a 3-point Likert scale (always = 2, sometimes = 1, and never = 0).

After ethical approval was obtained from the Ministry of Health and KAUH, a link to the online self-administered questionnaire was sent to the email addresses of the head nurses in each ward/unit meeting the inclusion criteria in the affiliated hospitals. The email clearly and precisely set out all relevant information about this study. Next, the head nurse was asked to send the link to the online self-administered questionnaire to all the nurses in the affiliated hospital’s wards/units who met the inclusion criteria. Lastly, due to the timeline, data analyses were started once 240 responses to the self-administered questionnaire had been received.

All obtained data were confidential and anonymous. Privacy was confirmed for all participants. The cover page of the questionnaire displayed a request for online informed consent while also explaining the purpose of this study and emphasizing the voluntary nature of participation.

The results showed that the Cronbach’s alpha for the questionnaire was from 0.890 to 0.960, and the general reliability for the questionnaire was 0.934, which means that the questionnaire has high reliability (Table 1).

### 2.4. Data Analysis

Data were coded using Microsoft Excel and analyzed through SPSS version 24.

Descriptive statistics (frequencies, percentages, means, and standard deviations) were used to describe categorical and quantitative variables. Responses to DVT knowledge and practice items were converted into percentages of correct knowledge and practice. The mean percentages of knowledge and practice scores were compared in relation to the demographic and professional characteristics of the nurses using Student’s *t*-test for independent samples and one-way analysis of variance followed by a multiple comparison test. Karl Pearson correlation coefficient and Spearman’s correlation was used to observe the correlation between the knowledge and practice scores and also between years of experience. Pearson’s chi-square test was used to observe the association between the knowledge–practice alignment (Yes and No) and the demographic and professional characteristics of the study participants. The reliability (internal consistency) of the questionnaire was assessed using Cronbach’s alpha. A *p*-value of ≤0.05 was used to indicate statistically significant results.

### 2.5. Ethical Consideration

Ethical approval for data collection was granted by the ethics committee of the Faculty of Nursing at King Abdulaziz University, Jeddah; by King Abdulaziz University Hospital; and by the Ministry of Health Studies and Research General Department. Then, permission was obtained from the nursing administration of King Abdulaziz University Hospital after the purpose of this study was explained. A research assistant from the MOH helped with contacting the nurses at the hospitals.

## 3. Results

Participants were asked 33 questions to assess their knowledge on the prevention of DVT, and they were categorized into two groups based on their mean scores. The results showed that more than the half of participants have an adequate level of knowledge on the prevention of DVT, with a total score of 75.64% ± 18.88; with regard to the domains, the highest level was observed for knowledge about the prevention and prophylaxis of DVT (81.98 ± 45.73%), followed by knowledge about the risk factors of DVT (74.12 ± 20.79%) and basic knowledge of DVT (72.01 ± 25.61%). The level of nursing practice for the prevention of DVT was 71.92%, with a mean score of 18.7. There were significant differences in the participants’ knowledge level based on different academic qualifications, their current ward, whether the participants had attended any training on DVT prevention, and whether they had read any professional research on DVT prevention, but there were no significant differences in knowledge level according to sex, current hospital, or whether there was a protocol/guideline in place for the prevention of DVT at the hospital. Similarly, there were significant differences in the practice level based on the participants’ current academic qualifications, their current ward, and whether the participants had attended any training on DVT prevention, but there were no significant differences in practice level according to sex, current hospital, whether the participants had read any professional research on DVT prevention, or whether there was a protocol/guideline for the prevention of DVT in place at the hospital.

### 3.1. Demographics, Work, and Academic Qualifications

The results showed that of the 240 participants with mean age of 35.15 years, most (84%) were female. Regarding their current academic qualifications, most of them (48.5%) had a bachelor’s degree; regarding their place of work, 51.2% were working in Ministry of Health hospitals and 48.8% were working at King Abdulaziz University Hospital. The mean years of experience of the study participants was 10.26 years.

However, regarding the participants’ current ward, the highest proportion (19.2%) worked in an ICU, while the remaining participants were employed in medical, surgical, emergency, outpatient clinic, CCU, OB-GYN, and other wards. Moreover, 60% had attended training on DVT prevention, 79.2% of the participants had read professional research on DVT prevention, and 97.5% said that there is a protocol/guideline for the prevention of DVT in place at their hospital (Table 2).

### 3.2. Nurses’ Level of Knowledge Regarding DVT Prevention

#### 3.2.1. Basic Knowledge of DVT

The results showed that the overall level regarding basic knowledge of DVT was 72.01%; the item about which respondents were most knowledgeable was “DVT occurs most frequently in the veins of the lower extremities”, as 90.83% of the nurses answered it correctly; and the item with which respondents were least familiar was “Deep vein thrombosis also occurs frequently in the upper limbs”, as 61.25% of the nurses were familiar with it (Table 3).

#### 3.2.2. Knowledge About the Prevention and Prophylaxis of DVT

The results showed that the nurses’ knowledge level regarding the prevention and prophylaxis of DVT was 81.98%. The item about which the nurses were most knowledgeable (91.67%) was “Elastic compression stockings may prevent DVT development”. The item about which respondents were least knowledgeable was “Bed rest is necessary after major surgery to prevent DVT”, as 66.25% of nurses answered it correctly (Table 3).

#### 3.2.3. Knowledge About the Risk Factors of DVT

The results showed that 74.12% of the nurses have adequate knowledge about the risk factors of DVT. The item about which the nurses were most knowledgeable (89.58%) was “Major surgery may predispose to DVT”. This was followed by “Prolonged immobilization predisposes to DVT in hospitalized patients”, which 87.5% of the nurses were familiar with, and 86.25% were aware of the item reading “Obesity may predispose to DVT”.

In contrast, the item about which the respondents were least knowledgeable was “Indwelling intravenous devices such as central venous catheters may predispose to DVT”, as 65% of nurses were aware of this. This was followed by “Low body mass index may predispose to DVT”, which 58.75% of the nurses knew about, and “Alcohol may predispose to DVT”, of which only 17.92% of the nurses were aware (Table 3).

#### 3.2.4. Differences in Mean Knowledge Percentage in Relation to Study Variables

There were significant differences in knowledge based on the nurses’ current wards, with those working at a surgical ward having the highest mean percentage of correct knowledge (83.2% ± 14.39) when compared with nurses working at other wards. The pairwise comparison test indicated that the nurses who were working in outpatient clinics had a significantly lower mean percentage of correct knowledge when compared with the nurses who were working at other wards. Also, the mean percentage of correct knowledge among nurses who had a master’s qualification was significantly higher when compared with nurses holding other levels of qualifications (diploma and bachelor’s degree). Also, there was a highly statistically significant difference in mean knowledge percentage between nurses who had attended training about DVT prevention (79.44% ± 18.58) and those who had not (69.95% ± 17.96) (*p*-value < 0.001), as well as between nurses who had read professional research on DVT prevention (78.07% ± 18.9) and those who had not (66.42% ± 15.86) (*p*-value < 0.001) (Table 4).

### 3.3. Nurses’ Practices Regarding DVT Prevention

#### 3.3.1. Nursing Practice in the Prevention of DVT

The results showed that the level of nursing practice in preventing DVT was 71.92%, with a mean score of 18.7. The practice items that were most frequently answered correctly were “Assessing the patients regularly for signs and symptoms of DVT/VTE”, with a mean score of 1.62, and “Educating the patients to avoid injury”, with a mean score of 1.60.

These were followed by “Encouraging patients to elevate their legs” and “Educating the patients on sufficient fluid intake”, each having a mean score of 1.58. The practice items with the lowest rates of correct answers were “Monitoring the side effects of the anticoagulants”, with a mean of score 0.75, and “Assessing the DVT risks of patients regularly”, with a mean score of 0.69 (Table 5).

#### 3.3.2. Differences in Practice Level According to the Main Variables

The results regarding the nurses’ practice showed a significant difference in practice level based on their current academic qualifications, where the practice level for those with a master’s degree (17.74% ± 7.89) was higher than that of those with a bachelor’s degree (19.23% ± 6.55), which in turn was higher than that of those with a diploma (17.74% ± 7.89) (*p*-value = 0.005). Nurses who had attended training about DVT prevention had a practice level of 19.57% ± 7.2, and those who had not had a practice level of 17.49% ± 7.2 (*p*-value = 0.029) (Table 6).

### 3.4. Correlation Between the Three Components of DVT Knowledge Scores and DVT PRACTICE SCORES

There were statistically significant positive correlations between (i) knowledge about DVT and DVT practice scores (*r* = 0.369, *p* < 0.0001); (ii) prevention and prophylaxis of DVT and DVT practice scores (*r* = 0.366, *p* < 0.0001); (iii) risk factors for DVT and DVT practice scores (*r* = 0.443, *p* < 0.0001); and (iv) total knowledge of DVT and DVT practice scores (*r* = 0.484, *p* < 0.0001).

### 3.5. Factors Associated with Better DVT Knowledge–Practice Alignment

A binary variable including the alignment between DVT knowledge and practice scores was created. That is, one group included nurses who had adequate knowledge and good practice scores (≥50% of correct scores for both knowledge and practice) and was termed the alignment category, whereas nurses who had scored <50% correct scores for both knowledge and practice were termed the non-alignment category. Out of the 240 subjects, 184 (76.7%) achieved ≥50% correct scores for both knowledge and practice, and 56 (23.3%) achieved <50% correct scores for both knowledge and practice. Bivariate analysis showed no statistically significant association between better DVT knowledge–practice alignment and age, sex, marital status, academic qualifications, working experience, and hospital. Also, there was a highly statistically significant association between better DVT knowledge–practice alignment and the nurses’ working ward (χ^2^-value = 35.01, *p* < 0.0001). That is, nurses who work in emergency, ICU, medical, OB-GYN, and surgical wards had higher scores (84.6%, 80.4%, 87.8%, 80%, and 82.9%) for both DVT knowledge and practice when compared with nurses working in the outpatient clinic (27.3%).

## 4. Discussion

This study aimed to assess the knowledge and practice levels of nurses regarding DVT prevention. The results revealed that the nurses’ knowledge level was 75.64%, with a 95% CI of ±18.88, and their practice level was 71.92%, with a mean score of 18.7. Interestingly, the highest knowledge level was related to the prevention and prophylaxis of DVT (81.98%), followed by knowledge about its risk factors (74.12%) and basic knowledge of DVT (72.01%). Significant differences in knowledge were observed between nurses with varying academic qualifications, with those holding doctoral, master’s, and bachelor’s degrees demonstrating higher knowledge than those with a diploma (*p*-value < 0.001). Furthermore, nurses working in surgical wards (83.2%) exhibited the highest level of knowledge, while ICU nurses had the lowest (74.97%).

These findings align with previous research, where similar educational disparities in knowledge were reported. A study conducted in northwest Ethiopia found a lower knowledge level (55.6%) than this study, possibly due to differences in sample size and regional factors affecting training opportunities [17]. Likewise, the level of knowledge in this study was higher than that reported by another study (59.90 ± 15.63%) [18]. However, the results of this study show a lower knowledge level compared to a study reporting 95.3% knowledge on DVT prevention [3], possibly due to differences in sample sizes or healthcare systems between the studies. The variation in knowledge levels based on ward type is also consistent with prior findings. For instance, surgical nurses in this study had better knowledge than ICU nurses, which corresponds with results from other studies where surgical nurses were found to have better DVT prevention knowledge compared to their ICU counterparts [19].

Regarding nursing practice, this study demonstrated that nurses with higher academic qualifications (master’s degree) exhibited better practices compared to those with bachelor’s or diploma degrees (*p*-value = 0.0005). Additionally, nurses working in surgical wards had the best practices, followed by those in medical wards, OB-GYN, and other wards. Nurses who had received training in DVT prevention showed better practices (19.57% ± 7.2) compared to those who had not (17.49% ± 7.2) (*p*-value = 0.029). These findings highlight the importance of continuous education in improving nursing practices. Similar results were found in other studies. For instance, nurses in China exhibited a practice level of 68.54% [20], while a study in India reported poor DVT prevention practices (86%) among nurses [21].

This study showed no significant relationship between knowledge and practice and sociodemographic factors, which is consistent with another study in Egypt, where the majority of nurses demonstrated poor practices despite having some level of knowledge [22]. This could suggest that knowledge alone does not necessarily translate into better practices, emphasizing the need for consistent training and practical implementation. However, in this study, there was a significant correlation between higher academic qualifications and improved knowledge, supporting the findings of another study that suggested a positive impact of advanced education on nurses’ knowledge [23].

The role of education in improving nurses’ compliance with DVT prevention guidelines is underscored in several studies. A study conducted in Australia showed that nurse education significantly improved compliance with VTE risk assessments and prevention guidelines [24]. Another study reinforced the importance of teaching evidence-based clinical practices to enhance the effectiveness of DVT prevention [5].

In conclusion, while our study found a relatively high level of knowledge and moderate practice level regarding DVT prevention among nurses, further improvements are needed, particularly through continuous education and training programs. Future studies should also focus on implementing strategies to bridge the gap between knowledge and practice, thereby improving patient outcomes in DVT prevention.

One limitation of this study is that self-administered questionnaires are prone to social desirability and recall bias. It is possible that the participants were prone to such biases. Although the present research cannot rule out this possibility, it seems helpful to point out issues that may conflict with these results. Another significant limitation that future studies could address is the inability to obtain the estimated sample size of 363; this was due to the time limits, since this study was an academic requirement for graduation purposes. However, to maintain the integrity of the findings of this study, this recruitment challenge was carefully taken into consideration during the analysis stage by obtaining this study’s power with the final sample size, which guarantees that the findings are still reliable and significant.

## 5. Conclusions

This study found that nurses in Jeddah, Saudi Arabia, demonstrate adequate knowledge and practices in DVT prevention, though fluctuations in responses suggest potential gaps in consistent prevention efforts. Factors such as higher education, formal training, and experience in surgical wards were linked to better knowledge. Ongoing education and risk assessment are recommended to enhance nursing practices and improve patient outcomes. Further research is needed to evaluate training effectiveness and identify strategies to strengthen nurses’ qualifications in DVT prevention.

## Figures and Tables

**Table 1:** Questionnaire reliability.

No.	Domains	No. of Items	Cronbach’s Alpha
1	The knowledge level of nurses on the prevention of DVT	33	0.890
2	Nursing practice in the prevention of DVT	13	0.960
	Total questionnaire	46	0.934

**Table 2:** Distribution of demographic and professional characteristics of study participants.

Variables	Categories		N	%
Sex	Male		37	15.4
	Female		203	84.6
Current academic qualifications	Diploma		103	43.1
	Bachelor’s		116	48.5
	Master’s		19	7.9
	Doctorate		1	0.4
Current hospital you work at?	In Ministry of Health hospitals		123	51.2
	King Abdulaziz University Hospital (KAUH)		117	48.8
Current ward you work in?	CCU		20	8.3
	Emergency		26	10.8
	ICU		46	19.2
	Medical ward		41	17.1
	OB-GYN		15	6.3
	Outpatient clinic		22	9.2
	Surgical ward		35	14.6
	Other		35	14.6
Have you ever attended any training about DVT prevention?	Yes		144	60
	No		96	40
Did you ever read any professional literature on DVT prevention?	Yes		190	79.2
	No		50	20.8
Is there any protocol/guideline in the hospital for prevention of DVT?	Yes		234	97.5
	No		6	2.5
	Minimum	Maximum	Mean	SD
Age	22	55	35.15	7.163
Experience	1	35	10.26	6.198

**Table 3:** Distribution of nurses’ responses regarding their knowledge about DVT, risk factors, and prevention.

	Items	True/False	%
1	DVT occurs as a result of stasis of blood (venous stasis); vessel wall injury; and altered blood coagulation.	True *	89.17
2	Venous thromboembolism (VTE) is a fatal complication of DVT.	True *	87.08
3	DVT occurs most frequently in the veins of the lower extremities.	True *	90.83
4	There is no relationship between cancer or cancer treatment and DVT/VTE.	False *	47.08
5	There is no relationship between respiratory diseases and DVT.	False *	56.67
6	Deep vein thrombosis also occurs frequently in the upper limbs.	False *	61.25
7	Foot and leg exercises may prevent DVT.	True *	90.00
8	Elevating legs is necessary to prevent DVT/VTE.	True *	78.75
9	Early ambulation after surgery may prevent DVT development.	True *	87.08
10	Bed rest is necessary after major surgery to prevent DVT.	False *	66.25
11	Heparin or low molecular weight heparin (LMWH) may prevent DVT development.	True *	91.25
12	Fluid restriction is necessary to prevent DVT.	False *	66.67
13	Elastic compression stockings may prevent DVT development.	True *	91.67
14	The use of intermittent pneumatic compression devices may prevent DVT development.	True *	84.17
15	Prolonged immobilization predisposes to DVT in hospitalized patients.	True *	87.50
16	VTE is a major cause of sudden death in hospitalized patients.	True *	72.50
17	Surgical patients are more prone than medical patients to DVT/VTE.	True *	78.75
18	Indwelling intravenous devices such as central venous catheters may predispose to DVT.	True *	65.00
19	Paralysis, paresis, or recent plaster cast on lower extremities may predispose to DVT.	True *	83.33
20	Obesity may predispose to DVT.	True *	86.25
21	Low body mass index may predispose to DVT.	False *	58.75
22	Advancing age may predispose to DVT.	True *	75.00
23	Previous DVT/VTE history may predispose to DVT.	True *	85.42
24	Major surgery may predispose to DVT.	True *	89.58
25	Varicose veins may predispose to DVT.	True *	83.33
26	Exercise may predispose to DVT.	False *	70.83
27	Trauma may predispose to DVT.	True *	79.58
28	Smoking may predispose to DVT.	True *	71.76
29	Alcohol may predispose to DVT.	True *	17.92
30	Cardiac diseases may predispose to DVT.	True *	81.67
31	Infections or inflammations may predispose to DVT.	True *	70.00
32	Pregnancy or post-partum may predispose to DVT.	True *	84.58
33	Oral contraceptives or hormone replacement therapy may predispose to DVT.	True *	66.67
	Total level		75.64

* Correct answer.

**Table 4:** Comparison of mean values of DVT knowledge percentage in relation to the study variables.

Variables	Categories	Mean	SD	Statistics	*p*-Value
Sex	Male	78.95	22.99	1.150	0.247
	Female	75.04	18.03		
Current academic qualifications	Diploma	70.61	19.73	6.724	<0.0001
	Bachelor’s	77.61	17.84		
	Master’s	88.84	9.74		
	Doctorate	93.94	.		
Current hospital you work at?	Within Ministry of Health hospitals	75.68	22.26	0.34	0.973
	King Abdulaziz University Hospital (KAUH)	75.6	14.6		
Current ward you work in?	CCU	79.85	15.86	7.616	<0.0001
	Emergency	78.09	20.52		
	ICU	74.97	16.49		
	Medical ward	77.16	13.77		
	OB-GYN	76.57	22.52		
	Outpatient clinic	51.24	24.85		
	Surgical ward	83.2	14.39		
	Other	77.92	14.39		
Have you ever attended any training about DVT prevention?	Yes	79.44	18.58	3.928	<0.0001
	No	69.95	17.96		
Did you ever read any professional literature on DVT prevention?	Yes	78.07	18.9	4.001	<0.001
	No	66.42	15.86		
Is there any protocol/guideline in the hospital for prevention of DVT?	Yes	75.89	18.8	1.247	0.214
	No	66.16	21.55		

**Table 5:** Distribution of nurses’ responses regarding the practices of DVT prevention.

No.	Items	Never	Sometimes	Always	Mean	SD
1	Providing information to patients and/or relatives about risks and prevention of DVT.	22	66	152	1.54	0.66
		9%	28%	63%		
2	Encouraging patients to perform foot and leg exercises by themselves or with relatives’ help if patients are unable to do so.	34	44	162	1.54	0.73
		14%	18%	68%		
3	Encouraging early ambulation of patients after surgery.	35	30	175	1.58	0.73
		15%	13%	73%		
4	Assessing the DVT risks of patients regularly.	74	166	0	0.69	0.46
		31%	69%	0%		
5	Administering anticoagulants as preventive in clinic.	35	43	162	1.53	0.74
		15%	18%	68%		
6	Monitoring the side effects of the anticoagulants.	60	180	0	0.75	0.43
		25%	75%	0%		
7	Educating the patients on anticoagulants.	27	30	183	1.65	0.67
		11%	13%	76%		
8	Educating the patients to avoid injury.	30	37	173	1.60	0.70
		13%	15%	72%		
9	Encouraging patients to elevate legs.	30	42	168	1.58	0.71
		13%	18%	70%		
10	Educating the patients on sufficient fluid intake.	36	29	175	1.58	0.74
		15%	12%	73%		
11	Using graduated compression stockings.	33	48	159	1.53	0.73
		14%	20%	66%		
12	Teaching the patients about proper use of graduated compression stockings.	33	39	168	1.56	0.72
		14%	16%	70%		
13	Assessing the patients regularly for signs and symptoms of DVT/VTE.	31	29	180	1.62	0.70
		13%	12%	75%		
14	Total level				18.7	7.25

**Table 6:** Comparison of mean values of practice percentage in relation to the study variables.

Variables	Categories	Mean	SD	Statistics	*p*-Value
Sex	Male	19.03	7.52	0.263	0.792
	Female	18.68	7.22		
Current academic qualifications	Diploma	17.74	7.89	4.427	0.005
	Bachelor’s	19.23	6.55		
	Master’s	21.89	5.82		
	Doctorate	0	.		
Current hospital you work at?	Within Ministry of Health hospitals	18.82	6.9	0.182	0.855
	King Abdulaziz University Hospital (KAUH)	18.65	7.63		
Current ward you work in?	CCU	19.25	8.12	4.585	0.000
	Emergency	19.19	6.63		
	ICU	19.46	7.47		
	Medical ward	19.61	6.13		
	OB-GYN	19.53	6		
	Outpatient clinic	11.18	7.13		
	Surgical ward	21.06	5.58		
	Other	18.23	7.77		
Have you ever attended any training about DVT prevention?	Yes	19.57	7.2	2.193	0.029
	No	17.49	7.2		
Did you ever read any professional literature on DVT prevention?	Yes	19.13	7.31	1.624	0.106
	No	17.26	6.91		
Is there any protocol/guideline in the hospital for prevention of DVT?	Yes	18.81	7.21	0.428	0.322
	No	15.83	9.22		

## Data Availability

Data are available upon reasonable request from the corresponding author.

## Public Involvement Statement

There was no public involvement in any aspect of this research.

## Guidelines and Standards Statement

This manuscript was drafted in accordance with the STROBE Statement for cross-sectional research.
